# Recent Perspectives Regarding the Role of Dietary Protein for the Promotion of Muscle Hypertrophy with Resistance Exercise Training

**DOI:** 10.3390/nu10020180

**Published:** 2018-02-07

**Authors:** Tanner Stokes, Amy J. Hector, Robert W. Morton, Chris McGlory, Stuart M. Phillips

**Affiliations:** Department of Kinesiology, McMaster University, Hamilton, ON L8S 4L8, Canada; stokest@mcmaster.ca (T.S.); hectoraj@mcmaster.ca (A.J.H.); mortonrw@mcmaster.ca (R.W.M.); mcglorc@mcmaster.ca (C.M.)

**Keywords:** skeletal muscle, protein turnover, resistance exercise, muscle hypertrophy, energy balance, energy restriction, amino acids

## Abstract

Skeletal muscle supports locomotion and serves as the largest site of postprandial glucose disposal; thus it is a critical organ for physical and metabolic health. Skeletal muscle mass is regulated by the processes of muscle protein synthesis (MPS) and muscle protein breakdown (MPB), both of which are sensitive to external loading and aminoacidemia. Hyperaminoacidemia results in a robust but transient increase in rates of MPS and a mild suppression of MPB. Resistance exercise potentiates the aminoacidemia-induced rise in MPS that, when repeated over time, results in gradual radial growth of skeletal muscle (i.e., hypertrophy). Factors that affect MPS include both quantity and composition of the amino acid source. Specifically, MPS is stimulated in a dose-responsive manner and the primary amino acid agonist of this process is leucine. MPB also appears to be regulated in part by protein intake, which can exert a suppressive effect on MPB. At high protein doses the suppression of MPB may interfere with skeletal muscle adaptation following resistance exercise. In this review, we examine recent advancements in our understanding of how protein ingestion impacts skeletal muscle growth following resistance exercise in young adults during energy balance and energy restriction. We also provide practical recommendations for exercisers who wish to maximize the hypertrophic response of skeletal muscle during resistance exercise training.

## 1. Introduction

Maintaining skeletal muscle mass throughout the lifespan is critical for the preservation of metabolic health and independent locomotion. While central to the production of contractile force, skeletal muscle also serves as the primary site of postprandial glucose disposal [1] and is the largest contributor to resting energy expenditure [2]. In addition to the health-centric importance of maintaining skeletal muscle there is also great interest, particularly within the athletic community, in enhancing the adaptive response of skeletal muscle to exercise training (i.e., improved force production and increasing muscle size: hypertrophy) with the aim of maximizing physical performance in competitive events. Thus, strategies to augment skeletal muscle hypertrophy and promote optimal remodeling and reconditioning of skeletal muscle following exercise training is an intense area of scientific inquiry with ramifications in both the clinical and athletic settings.

There is now a wealth of studies that have characterized the response of skeletal muscle to changes in nutritional and contractile stimuli. What these studies have shown is that the size of skeletal muscle is dependent upon the kinetic processes of muscle protein synthesis (MPS) and muscle protein breakdown (MPB), the algebraic difference (MPS minus MPB) between which dictates net protein balance (NPB). When diurnal fluctuations in MPS equal those of MPB, muscle mass is maintained. Muscle protein accretion leading to the growth of muscle fiber size, is only achieved when net rates of MPS exceed MPB and NPB is positive. Alternatively, net muscle catabolism (atrophy) leading to the loss of muscle proteins, occurs when MPB exceeds MPS and NPB is negative [3,4]. Increased loading of skeletal muscle and hyperaminoacidemia following dietary protein intake, independently and synergistically exert a positive influence on NPB by modulating the relative balance between MPS and MPB [3,4]. Indeed, in the postabsorptive state, an acute bout of resistance exercise stimulates MPS by more than 100% above basal levels [5]; however, NPB remains negative due to the concomitant activation of MPB. Only when protein is ingested following a resistance exercise bout is there a synergistic impact on MPS resulting in a protracted state of positive NPB [6]. Repetitive bouts of resistance exercise in combination with protein intake increase NPB and promote muscle protein accretion over time.

In this brief review, we focus on how dietary protein is utilized in support of skeletal muscle protein remodeling and how protein may facilitate increased post-exercise MPS and ultimately impact hypertrophy. To gain a deeper understanding of this concept, we address the human capacity to digest protein and contrast that with the ability of skeletal muscle to utilize the available amino acids for MPS. In addition, we discuss the issue of the per meal quantity of protein that optimally stimulates daily MPS and speculate as to why a strategy focused on persistent suppression of MPB may be ill-suited to the goal of promoting muscle hypertrophy with resistance exercise training. Leveraging large sample sizes from recent meta-analyses we attempt to provide an ‘optimal’ prescription for protein intake for maximizing protein remodeling and hypertrophy following resistance exercise. Finally, we provide a brief discussion of recent findings regarding strategies to maintain or even increase skeletal muscle mass during periods of energy restriction.

## 2. Understanding the Limits to Muscle Protein Synthesis: How Much Protein Can Muscle Use?

**Figure 1:**
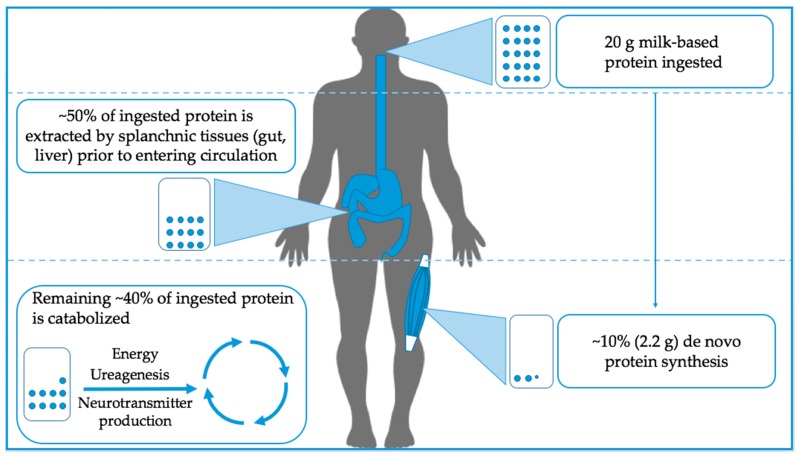
Simplified overview of whole body oral protein utilization at rest. Of the protein ingested, approximately 50% is extracted by splanchnic tissues before entering peripheral circulation. Interestingly, only ~10% of the ingested protein is utilized for skeletal muscle protein synthesis while the rest is catabolized.

The capacity to digest and absorb dietary protein and the subsequent aminoacidemia far exceeds the capacity of skeletal muscle to utilize the constituent amino acids for the purpose of muscle anabolism. Following oral ingestion, protein digestion is initiated in the stomach by pepsin in the presence of hydrochloric acid and continues in the duodenum by the secretion of pancreatic proteases and enterocyte proteases. The end products include peptide fragments and free amino acids that are absorbed almost exclusively in the small intestine. The gut is a highly metabolically active organ [7,8], and extracts ~40%–50% of the available amino acids from the ingested protein meal primarily for the purposes of energy production and for local protein synthesis [9]. The remainder (~50%) of amino acids is released into the hepatic portal vein prior to being taken up by the liver. Like the gut, the liver utilizes amino acids for local metabolism but, rather than primarily oxidizing amino acids, a significant proportion of amino acids are instead used for the synthesis of hepatic and liver-derived blood proteins [10]. The amino acids that have been sequestered by the splanchnic tissues and the liver are ‘first-pass cleared’ and thus are not available for peripheral metabolism. It is interesting to note that the branched-chain amino acids (BCAAs), which are implicated in skeletal muscle anabolism [11], are catabolized to a relatively minor extent by the liver due to a low content of the branched-chain aminotransferase enzyme in human hepatocytes [12,13]. A disproportionate amount (relative to the composition of ingested protein) of amino acids released from the splanchnic bed into the hepatic vein are therefore BCAAs [14]. Overall, ~50% of the amino acids in a protein-containing meal are extracted by the splanchnic tissues whereas the rest are released into the plasma circulation for extra-splanchnic utilization [15]. Although skeletal muscle is a large depot for the retention of amino acids, not all the amino acids released into plasma are destined to become incorporated into new skeletal muscle tissue. In a recent study employing an intrinsically-labeled tracer approach, Groen and colleagues [15] demonstrated that only ~2.2 g or 11% of the amino acids provided to young men in a 20 g bolus of casein protein were used for de novo protein synthesis despite ~55% availability in the peripheral circulation following splanchnic extraction. The remaining amino acids are catabolized and serve as substrates for a range of metabolic processes from energy production and urea synthesis and, to a very minor extent, neurotransmitter production (see Figure 1 for an overview). It is, however, important to acknowledge that states of disease as well as age may alter the kinetics of amino acid metabolism following protein ingestion [16,17]. Future research should focus on determining how factors such as protein type [18], age [16], and potentially the gut microbiota [19] interact to influence amino acid partitioning and specifically how these factors change in the context of resistance exercise.

Lower quality proteins, such as soy or wheat protein, that lack or are low in one or more essential amino acids, fail to stimulate MPS to the same degree as higher quality sources [20,21] and the constituent amino acids could presumably become less enriched in muscle tissue at the same relative dose. Regardless of the protein source, the feeding-induced rise in plasma amino acid concentrations drives uptake across the muscle membrane. Some evidence suggests that protein ingestion induces gene expression of several amino acid transport proteins [22,23] that may increase the influx of amino acids into skeletal muscle. Following substantial hyperaminoacidemia, there is a ~30 min delay in the stimulation of MPS before it peaks at 2 h [24,25]. Importantly, aminoacidemia-induced activation of MPS is transient and MPS reverts to basal levels after ~2–3 h despite continuing hyperaminoacidemia [25]. This phenomenon, which has been corroborated in studies employing both infusion of amino acids [26] and oral bolus protein ingestion [27], has been coined the ‘muscle-full’ effect and explains why simply consuming protein in the absence of contractile activity fails to induce protein retention and skeletal muscle hypertrophy. The stimulation of MPS in response to hyperaminoacidemia appears to be entirely driven by the essential amino acids contained within protein [28], and of these amino acids, leucine is the primary amino acid agonist [29,30,31]. While it is true that leucine is capable of stimulating MPS in the absence of other amino acids, it should be emphasized that protein synthesis will eventually become limited by the availability of other essential amino acids. However, enriching a lower quality protein with leucine, provided a full complement of essential amino acids is also present, may induce a comparable stimulation of MPS to that seen in higher quality sources [32]. Regardless, that fact that MPS plateaus despite sustained hyperaminoacidemia suggests that MPS is a saturable process and that amino acids above certain quantities provide no further stimulation. In this regard, Moore et al. [33] showed that 0.24 g/kg (±0.06 g/kg; 95% confidence interval (CI)) body mass of protein maximally stimulated rates of MPS in younger males, but ~0.40 g/kg (±0.19 g/kg; 95% CI) body mass of protein is required in older adults to achieve a comparable stimulation of MPS: 0.056%/h vs. 0.058%/h in older and younger adults, respectively. Thus, per meal doses would be recommended to be ~68% higher in older persons to achieve similar levels of MPS [33].

**Figure 2:**
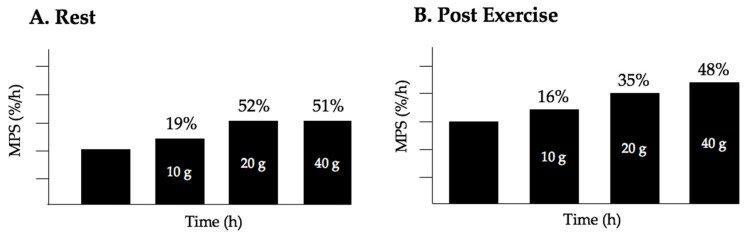
Whey protein ingestion-induced increase in MPS in young men, percent change from 0 g. (**A**) At rest, consumption of 10 g or 20 g of protein results in a rise of 19% and 52% respectively from 0g. Consumption of 40 g of whey protein does not result in superior stimulation of MPS beyond consumption of 20 g; (**B**) Following resistance exercise, consumption of 20 g of protein increases MPS almost twice as much as consumption of 10 g, while consumption of 40 g of whey protein results in a small stimulation of MPS over and above that seen at 20 g indicating there are diminishing returns in terms of stimulation of MPS above 20 g. Data redrawn from Witard et al. [35], however, similar data are reported by MacNaughton et al. [38], and Moore et al. [36].

We know that exercise sensitizes the muscle to hyperaminoacidemia [34], which suggests that habitual exercise would shift the dose-response curve to the left and lower intakes of protein would be needed to stimulate MPS; however, it may be that exercise also increases the capacity for use of amino acids so the maximal gain in MPS may require higher intakes of protein. With respect to recovery from exercise, Witard and colleagues [35] employed a unilateral exercise model to probe for differences between rested and exercised-stimulated rates of MPS and made similar observations as Moore et al. [36] when measuring myofibrillar-specific protein synthesis in young men administered titratable doses of whey protein (see Figure 2 for the relative increase in MPS at each dose of protein). Importantly, Moore [36] and Witard [35] both observed that protein doses beyond ~20 g (equivalent to ~0.24 g/kg body mass per meal) resulted in a negligible further stimulation of MPS, such that 40 g of protein provided no statistically significant enhancement in rates of MPS either at rest or following resistance exercise. Instead, as would be expected, leucine oxidation increased when protein dose exceeded 20 g [36], or there was enhanced conversion of phenylalanine to tyrosine [35], and an increase in plasma urea production and concentration [35]. It is important to note that biopsy acquisition time points in the aforementioned investigations [35,36] only permitted MPS measurements over ~4 h period following exercise; however, cumulative MPS over the course of a day also influences the accretion of myofibrillar protein. Therefore, it may be more important to consider the protein dosing strategy that maximizes MPS during waking hours rather than at a single meal. Areta and colleagues [37] demonstrated that the intake of 20 g whey protein administered every ~3 h was more effective at stimulating MPS over a 12 h period following bilateral leg exercise than protein-equated doses administered as smaller, more frequent pulses (10 g every 1.5 h), or larger boluses consumed less frequently (40 g every 6 h). Given that the studies mentioned above [35,36,37] measured MPS using stable isotope infusions in a controlled laboratory setting, future research should replicate these studies under free-living conditions using deuterium oxide to capture the influence of normal daily eating patterns on MPS. Cumulatively, these findings suggest that ~20 g of high-quality protein (or ~0.3 g/kg/meal) is sufficient to maximally stimulate MPS after a single meal and, when repeatedly administered 3 h apart, optimize MPS throughout the day.

A recent two dose study performed by MacNaughton and colleagues [38] detected a statistically significant 19% greater stimulation of MPS following whole-body resistance exercise in young men consuming 40 g of protein compared to those consuming 20 g. To place this finding into context, the added stimulation observed with ingestion of 40 g compared to 20 g of protein by Moore and Witard (who also measured responses at lower and zero protein) was 11% and 13%, respectively. Given that the only salient difference between the experimental design employed by MacNaughton and those utilized by Moore and Witard was the exercise protocol (whole-body vs. unilateral), these data [35,36,38] suggest that young men performing whole-body resistance exercise may opt to ingest greater amounts of protein than those performing lower body exercise to maximize muscle anabolism. However, the incremental gain in muscle mass would appear to be marginal and likely of no great relevance to recreationally exercising individuals. Finally, since MacNaughton et al. only tested two protein intakes (20 g and 40 g) it is unknown whether an intake between these two levels (e.g., 30 g) could have resulted in a similar stimulation of MPS. In addition, it was not possible in this study [38] to determine a true dose response and thus determine the robustness of the small difference between MPS at 20 g and 40 g of protein. Recommended daily protein intakes are body mass-specific and heavier athletes will require quantitatively more protein than lighter athletes at each meal to fulfill their daily requirements. However, even a 100 kg athlete can satisfy his/her daily protein requirements by consuming ~30 g of protein at each meal, which aligns with the per meal recommendations of 0.24–0.30 g/kg (including upper 95th confidence interval) advocated by Moore et al. [33]. Thus, it is important to consider contextual variables such as body mass and exercise modality when advocating on optimal protein dosing strategies.

More recently, the ingestion of protein prior to sleep has garnered significant interest as it relates to the recovery from successive bouts of resistance exercise. Adults aged 18–64 years sleep on average for ~7 h each night, rendering the overnight period the longest postabsorptive period of the day if one considers that the last meal may be consumed 3–4 h prior to sleep. Until recently, little attention was given to the overnight sleeping period; however, in the context of stimulation of MPS, an optimal prescription for protein ingestion would include something that could offset negative NPB after overnight fasting. In a proof-of-principle study, Groen et al. [39] demonstrated that nasogastric administration of 40 g casein protein effectively stimulated MPS and improved whole-body protein balance over a 7 h overnight sleep. This finding is particularly relevant in athletes and recreationally active adults who may exercise in the evening hours and therefore consume protein supplements soon before they sleep. Limited evidence has shown that ingestion of ~20 g of protein, which maximizes MPS at each meal during waking hours, may be unable to enhance overnight muscle protein anabolism to a significant degree [40]. However, it is possible that the lack of effect of protein on overnight muscle anabolism observed by Beelen and colleagues [40] was due to a washout from the long period of tracer incorporation (9 h). The same group later demonstrated a significant 22% increase of overnight mixed MPS in young men who ingested 40 g of casein protein immediately prior to sleep [41]. The same dose consumed after exercise was also shown recently to stimulate overnight MPS 30% more than the same dose of protein consumed without the prior performance of resistance exercise [42]. This finding suggests that the exercise-induced sensitization of skeletal muscle to amino acid intake extends into the overnight recovery period [42,43]. Examined over a chronic period, pre-sleep ingestion of 27.5 g casein protein (as a hydrolysate-intact protein blend) augmented gains in quadriceps cross sectional area, type II fiber cross sectional area, and the sum of a several 1-RM assessments compared to those partaking in the same exercise protocol without pre-sleep protein ingestion [44]. It is important to note that, although Snijders and colleagues [44] did conclude that protein supplementation prior to sleep enhanced gains in quadriceps cross sectional area compared to placebo intake, there was no difference in lean body mass accrual between groups with resistance training. Cumulatively, these data [39,40,41,42,43,44] suggest that casein protein consumed prior to sleep represents an effective strategy to promote skeletal muscle anabolism, but that pre-sleep doses of protein may need to be somewhat higher than those recommended during the daytime, appearing to be in the range of ~30–40 g.

A recent meta-analysis and meta-regression conducted by our laboratory demonstrated that protein supplementation is sufficient to optimize resistance exercise training-induced gains in fat-free mass [45]. Indeed, Morton et al. [45] demonstrated that protein supplementation during resistance training for ≥6 weeks augmented lean mass accretion by 27% (~0.3 kg) on average, which is similar to a previously conducted meta-analysis (e.g., ~0.7 kg; [46]). In a breakpoint analysis, we also demonstrated that beyond a daily protein intake of 1.6 g/kg/day (1.0–2.2; 95% CI), protein supplementation failed to augment resistance exercise-induced muscle hypertrophy [45]. Although timing [37], dose [33,35,36,38], and source [20,21] may influence the efficacy of protein supplementation on MPS, our meta-analysis showed that these variables do not necessarily translate into enhanced muscle accretion in a chronic exercise setting [45]. Rather, a daily protein intake of ~1.6 g/kg/day or as high as 2.2 g/kg/day, appears to be the most influential factor to consider when optimizing muscle mass accretion with resistance exercise is the goal. This daily protein intake could be achieved via the incorporation of high-quality protein sources at each meal throughout the day and, if necessary, supplementing the diet with high-quality (i.e., whey or casein) protein supplements.

## 3. Protein-Induced Inhibition of Proteolysis: A Good Thing for Enhancing Muscle Protein Accretion?

Resistance exercise results in skeletal muscle damage that compromises the architectural integrity of the myofibril [47]. Damage may be induced mechanically, as evidenced by z-disk streaming [47,48], or can be manifested as increased protein carbonylation [49], indicative of exercise-induced oxidative stress. It is likely that the exercise-induced muscle damage stimulates an increase in MPB that is observed following resistance exercise [5]. From a physiological perspective, an acute increase in MPB is required to dismantle and repair damaged proteins and to help restore muscle function. In the postabsorptive state muscle proteolysis results in release of amino acids into the intramuscular free pool for subsequent use by the protein synthetic machinery (i.e., intracellular recycling), thereby supporting MPS in the absence of exogenous protein intake [50]. Theoretically, this remodeling response after successive bouts of resistance exercise serves as an effective mechanism to reduce subsequent exercise-induced damage. A recent study by Damas and colleagues [48] showed that an unaccustomed bout of resistance exercise in young men resulted in z-disk streaming and swelling-induced edema, indicating the presence of muscle damage. Moreover, rates of MPS were shown to be highest following the initial bout of an unaccustomed exercise bout than after completing the same bout of exercise following 3- and 10-weeks of resistance exercise training. Importantly, when the acute MPS response was normalized to the magnitude of z-disk streaming at each relative phase of the study, there were no detectable differences in MPS. This finding suggests that during the early resistance-training period, MPS is robustly stimulated to facilitate the repair and remodeling of exercise-induced protein damage. As the transition of muscle tissue from exercise-naïve to ‘resistance-trained’ occurs, MPS was adaptively reduced and directed more towards myofibrillar remodeling rather than a global remodeling all muscle protein fractions. Consequently, training attenuates the MPS response to resistance exercise [51,52]. Our laboratory has also shown that young men who performed unilateral resistance training for 8 weeks had a more pronounced stimulation of MPS in their trained limb relative to their untrained limb 4 h after exercise completion; however, MPS returned to basal levels 28 h after exercise only in the trained limb [53]. We propose that MPB follows a similar adaptive decline with resistance training characterized by a robust stimulation following the first bout of unaccustomed exercise to initiate global remodeling, followed by an adaptive decrease with training. Analogously, antioxidant enzyme content increases from an untrained state following resistance training [54], which in turn may buffer exercise-induced oxidative damage. Taken together, increased protein turnover appears to be necessary, especially during the early resistance-training period, to facilitate skeletal muscle remodeling and to lay the foundation for subsequent muscle protein accretion with progressive training. The impact of suppressing this normal rise in MPB is not known, however, it appears unlikely, in our estimation, to translate into any physiological benefit. Two nutritional interventions capable of suppressing MPB, thereby enhancing net protein balance, include increasing systemic insulin concentrations via carbohydrate ingestion, and/or by supplementing with higher doses of protein (than are needed to maximally stimulate MPS) [55,56].

In pre-clinical rodent models, insulin stimulates MPS [57], however, in humans insulin concentrations above ~5 IU/mL do not appear to stimulate MPS [58]. In a recent meta-analysis [59], it was concluded that insulin is merely permissive for the stimulation of MPS and predominantly regulates muscle anabolism through its inhibitory influence on MPB. Even moderate elevations of plasma insulin achievable by consuming a mixed-meal or protein beverage are sufficient to reduce MPB by ~50% [58,60], with no further suppression at insulin concentrations above ~30 mU/L. It is, therefore, not surprising that investigations pairing carbohydrate and protein intake post exercise have failed to observe a superior anabolic response compared to protein ingestion alone [61,62,63]. The triviality of carbohydrate in augmenting muscle anabolism was demonstrated by Staples et al. [62], who failed to detect a greater stimulation of MPS or a greater suppression of MPB when 50 g of carbohydrates were added to a dose of 25 g of whey protein when evaluated against 25 g of protein alone, despite enhanced anabolic signaling and a substantially greater insulinemia. Thus, carbohydrate coingestion with protein does not enhance the anabolic effect of protein and does not contribute to a greater hypertrophic potential following resistance exercise. However, given that protein is habitually ingested as a mixed-meal, it is prudent to mention that carbohydrates do not impair the anabolic response to protein, despite increasing splanchnic retention of amino acids and slowing the rate at which they enter the systemic circulation [64]. Moreover, ingesting carbohydrates restores muscle glycogen following exhaustive exercise [65,66], and may therefore be important for performance recovery in an athletic setting (for a recent review see Ref. [67]).

Amino acid intake at sufficiently high levels (~70 g) also appears to inhibit proteolysis in an insulin-independent manner [68]. In theory, by continuing to ingest amino acids beyond the point at which MPS is maximally stimulated (i.e., 20–30 g in healthy young adults), intracellular amino acid concentrations rise and inhibit MPB rather than further stimulating MPS and, by extension, promotes a greater NPB. Kim et al. [56] have suggested that solely focusing on MPS to generate recommendations on the protein dose necessary to optimize the anabolic response to a meal neglects the contribution that suppression of proteolysis would have on promoting a more positive NPB. They argue that, while data generated from their laboratory [69] corroborates the finding that ~25 g of high quality protein maximizes the MPS response at rest or following exercise, greater amounts of protein promote an incrementally greater whole body NPB, primarily by suppressing whole-body (note: MPB has not been measured) protein breakdown [68]. To provide experimental evidence to support their thesis, Kim et al. [68] recently compared the anabolic response of 40 g vs. 70 g of protein, in the form of beef patties, either at rest or following a bout of exhaustive resistance exercise on NPB. While muscle-specific protein synthesis was increased to a similar extent in both groups, NPB was more positive in the higher protein group due to a greater suppression of whole-body protein breakdown and a smaller, albeit significant, increase in whole-body protein synthesis. Given that skeletal muscle may constitute ~25% of whole-body proteolysis [70], it is possible that 70 g of protein suppressed MPB, however, such speculation requires further investigation. In another study [71], by the same group, it was demonstrated that a positive linear relationship existed between incrementally higher protein intakes (from ~6 g to 92 g) and whole body NPB that led the authors to conclude that no practical upper limit exists regarding the amount of protein that could maximize muscle anabolism; however, once again the responses measured were not muscle-specific.

While consumption of greater quantities of protein per meal than what we are recommending here (i.e., ~20–30 g/meal) may suppress proteolysis, we see little evidence to support strategies that aim to specifically suppress MPB following resistance exercise due to the role MPB would play in protein remodeling during recovery from exercise and because of our relative lack of understanding of the potential consequences of doing so. Indeed, rodent data provides a useful lens through which to view the potential physiological consequences of inhibiting proteolysis. The two predominant systems responsible for protein-specific and macromolecular breakdown are the ubiquitin-proteasomal (UPS) and autophagic-lysosomal systems, respectively. Knocking out Atg7 in rodents, a critical autophagy gene, results in a 40% decrease in myofiber size compared to wild-type littermates [72]. Moreover, Atg7 null mice have grossly impaired muscle specific force, likely resulting from z-disk misalignment acting in confluence with increased protein carbonylation [72]. Similar impairments have been observed in mice who have had TSC1 knocked out, consequently leading to sustained mTORC1 activation and a chronic inhibition of Ulk-1, an upstream activator of autophagy [73]. Lastly, rodents with the Rpt3 (an essential subunit in the 19S core of the 26S proteasome) gene conditionally knocked out, exhibited a reduced lifespan as well as smaller myofiber CSA, and severely impaired grip strength [74]. Taken together, animal work in combination with extant human data [5] strongly implicate an important role for basal rates of autophagy- and UPS-mediated protein breakdown in the maintenance of skeletal muscle viability. Admittedly, suppression of MPB by nutritional means is not synonymous with the suppression of MPB from the knockout of key proteolytic factors, and it is improbable that the consequences of suppressing MPB would be remotely characteristic of genetic ablation. However, there is cross-sectional evidence [75] to suggest that even moderate differences in the regulation of proteolysis are related to poor muscle quality and frailty. For example, a lower expression of genes involved in autophagy, mitophagy, and the UPS was shown to be associated with sarcopenia and impaired physical function, as indicated by lower lean mass and a reduced travel distance during a 6-min walk test in older women [75]. The thesis behind these data [75] is there is a decreased clearance of misfolded and/or aggregated proteins in individuals with lower autophagy flux, leading to lower quantity and quality of muscle.

In the context of resistance exercise, the role played by proteolytic factors is not as clear. A study by Leger et al. [76] demonstrated a 10-fold and 2.5-fold increase in MuRF1 and atrogin1 mRNA, respectively, following an 8-week resistance-training study. Importantly, atrogin1 protein content increased ~40%, despite the participants experiencing a 10% gain in quadriceps muscle size. Presumably, increased atrogin1 protein content constitutes one of a number of adaptations that occur to modulate skeletal muscle protein breakdown in the presence of increased contractile and structural protein content, rather than being involved in muscle atrophy per se. It is not clear what effect attempting to suppress, through dietary or other means, this exercise-induced increase in atrogin1 protein content, or an acute activation of MPB in general, would have on the integrity of skeletal muscle but if work in rodents is any indication, basal protein degradation is necessary to ensure optimal skeletal muscle function. Moreover, suppressing whole-body protein breakdown following exercise may result in transient organ tissue accretion (a component of lean body mass) rather than muscle hypertrophy per se, which cannot be easily discerned from DXA-derived lean body mass changes. More research is now needed to characterize the impact of mildly suppressing protein breakdown, both at the whole-body and skeletal muscle level, on the phenotypic adaptations to resistance exercise. However, in our view athletes and recreationally active individuals should focus on practices to maximize MPS rather than suppress MPB, which appears to be physiologically important for skeletal muscle remodeling following damaging exercise.

## 4. Protein Intake during Energy Restriction

When energy intake is not sufficient to balance energy expenditure, a decrease in total body mass occurs. These periods of energy restriction may be voluntarily invoked, as is the case for athletes competing in body mass-restricted events and competitions focusing on muscle aesthetics (i.e., bodybuilding), or it can be involuntary in nature, such as during military operations. In general, an energy deficit can be achieved via either, or a combination of, caloric restriction or a chronic elevation in physical activity levels (with no compensatory increase in energy intake [77]). In overweight and obese individuals, the ensuing body mass loss that follows a protracted energy deficit generally confers physical as well as metabolic health benefits such as improved hepatic and skeletal muscle insulin sensitivity, as well as improvements in beta-cell function [78]. However, a concerning consequence of energy-restriction is that lost weight is comprised, in general, of a 25% loss of lean body mass (LBM) [79], a significant proportion of which is skeletal muscle. This loss of skeletal muscle mass could subsequently lead to reductions in performance and increased injury susceptibility. Thus, regardless of the antecedent cause of the energy deficit, it may be important for athletes that efforts be made to ensure maintenance of LBM.

Evidence is mounting to suggest that a reduction in postabsorptive and postprandial MPS is contributing and may be the main adaptive mechanism driving the loss of LBM during energy restriction. Following a ~20% energy deficit, postabsorptive rates of MPS were found to be reduced by ~19% compared to measurements made during body mass maintenance [80]. This decrement in MPS was likely precipitated by reduced intramuscular anabolic signaling, as evidenced by a ~35% and 30% lower phosphorylation of Akt^Ser473^ and 4E-BP1^Thr37/46^, respectively [80]. Given that MPS is an energy-demanding process, the declines observed during caloric restriction likely represent a conservation-oriented mechanism to avoid the inefficient utilization of ATP for growth promoting processes when food availability is low. In line with this thesis, reductions in MPS occur rapidly following the onset of food deprivation. For example, Areta et al. [81] observed a ~27% reduction in myofibrillar protein synthesis after only 5 days of energy restriction in young men and women. As energy restriction is prolonged, the changes in MPS appear to plateau at a level suitable to the prevailing nutrient abundance [82].

Exactly how rates of MPB change in response to energy-restriction is less well characterized, particularly owing to the difficulty of applying methods currently available to quantify MPB. Carbone and colleagues [83] investigated the changes in molecular markers of MPB to infer the dynamics of muscle proteolysis in response to a ~40% energy-restricted diet. They failed to demonstrate any changes in 26S proteosomal proteolytic activity, but observed a 1.2- and 1.3-fold increase in MuRF1 and atrogin1 mRNA, respectively. These observations led the authors to speculate that MPB is elevated in response to energy restriction [83]. Later, the same group showed that fractional breakdown rate was elevated ~60% above basal levels after 10 days of energy deficit, with a small concomitant rise in caspase-3 [84] in a cohort of highly active cyclists. These findings are at odds with findings from our laboratory in response to a comparable dietary intervention. Hector et al. [85] exposed 24 overweight participants to a 10 d period of 40% caloric restriction (relative to energy requirements) and found no significant changes in MPB or any static marker of muscle proteolysis. The disparate observations of Carbone et al. [84] and Hector et al. [85] showing an elevated and an unchanged rate of MPB are difficult to reconcile; however, they may be due to the different study populations (active adults in Carbone et al. and overweight adults in Hector et al.). Nonetheless, we contend that there is a compensatory decrease in energy-consuming processes during periods of caloric restriction. Muscle proteolysis, which is accomplished primarily via UPS-mediated degradation, is heavily reliant on ATP to fuel the conjugation and ligation of ubiquitin molecules onto a target substrate [86]. Furthermore, the tight 19S cap of the 26S proteasomal complex prohibits folded proteins from entering the 20S catalytic core [86]. Each subsequent step of protein breakdown is therefore reliant on sufficient energy. Autophagy also plays a key role in macromolecular breakdown during energy-deprived conditions, however the scope of the current review precludes a detailed discussion in this regard. Given the high energy cost of MPB, we propose that it is unlikely that energy-restriction would increase MPB to the extent observed by Carbone et al. [84]. Taken together, efforts to combat the loss of LBM during periods of energy restriction would be most fruitful by employing strategies that minimize the decrements in MPS.

Strategies that have proven effective at maintaining or even augmenting LBM during periods of energy restriction include practicing loading exercise and, to a lesser extent, increasing daily protein intake. During energy balance, as we have discussed above, daily protein intakes of 1.6 g/kg/day maximize the hypertrophic potential of skeletal muscle following a resistance-training intervention [45]. Under energy-restricted conditions, however, a greater relative proportion of amino acids are catabolized for energy production, resulting in fewer amino acids available for muscle anabolism. It could be contended that by increasing protein intake during calorically restricted periods, energy production can be sustained while also preserving MPS. Indeed, Pasiakos et al. [87] measured MPS in response to protein-rich meal before and after 21 days of a 40% energy deficit, achieved by 30% caloric restriction in combination with a 10% increase in aerobic exercise. The authors demonstrated that only participants consuming 1.6 g/kg/day (twice the recommended dietary allowance for protein-RDA) and 2.4 g/kg/day (3 times the RDA) preserved their anabolic sensitivity to protein. In contrast, the group consuming the RDA (0.8 g/kg/day) for protein had a suppressed MPS response to protein ingestion after being in an energy deficit. The RDA-consuming group also lost substantially more total mass as fat-free mass compared to those consuming 2- or 3-times the RDA for protein [87]. Despite retaining anabolic sensitivity to a protein containing meal, even the participants consuming protein at levels greater than the RDA for protein experienced significant losses of LBM [87]. Taken together these data [87] suggest that protein intake alone is insufficient to maintain LBM during energy restriction.

Engaging in resistance exercise during an energy deficit has a sparing effect on LBM [81,85,88]. Indeed, LBM was retained following a 4-week energy-restricted diet in individuals who resistance trained 6 days/week and consumed 1.2 g/kg/day of protein [88], a daily protein intake that was hypothesized to be insufficient to prevent reductions in LBM in the absence of exercise [87]. Moreover, participants who consumed 2.4 g/kg/day protein (3-times the RDA) in conjunction with the same 6 day/week exercise regimen increased their LBM over the 4-week period [88]. Areta et al. [81] reported that resistance exercise restored the ~27% decrement observed in postabsorptive MPS during energy restriction, and when paired with protein ingestion, significantly enhanced MPS above values obtained in energy-balance. Using ingestion of deuterium oxide, we also demonstrated a preservation of integrated myofibrillar protein synthesis, which was only observed in participants consuming 2.4 g/kg/day protein [85]. It is important to note that we studied an overweight population and therefore these findings may not be applicable to leaner subjects undergoing periods of energy restriction who, despite higher intakes of protein, may be unable to retain LBM. A meta-analysis [89] demonstrated that leaner subjects with resistance-training experience were more vulnerable to losses in LBM than exercise-naive individuals with a higher body fat percentage. Thus, athletes who tend to be leaner than the general population, and who have more training experience, have been recommended to consume protein intakes upwards of ~3 g/kg/day in an attempt to prevent LBM losses during energy restriction [89]. It is important to note, however, that the protein intake necessary to offset reductions in LBM is likely more reliant upon the severity of energy restriction and the amount of resistance exercise habitually performed [90]. Nevertheless, resistance exercise when performed in conjunction with consumption of a higher protein intake is effective for mitigating losses and leading to maintenance or allowing LBM accrual, during energy restriction.

In addition to protein requirements it is also important to consider the satiating effect of protein. Many athletes who compete in aesthetically inclined sports (i.e., bodybuilding) begin dieting aggressively anywhere from 8–16 weeks before competition. These periods are often characterized by pronounced caloric deficits aimed at reducing body fat to very low levels, while retaining the greatest proportion of muscle mass possible [91]. The success of an athlete during this period is predicated on his/her ability to chronically adhere to a calorically restricted diet. Thus, maximizing the satiating effect of each meal is paramount during these protracted periods of energy restriction. Protein is the most satiating macronutrient [92,93] and should therefore be the cornerstone of any weight loss plan; however, not all protein sources modulate hunger and satiety levels to the same degree. Under acute settings, in which a breakfast composed primarily of whey protein was compared to casein protein, hunger was suppressed to a greater extent in participants consuming whey protein [94]. This effect may be partly due to the efficacy of whey protein to stimulate a more pronounced secretion of the satiety-inducing hormone glucagon-like peptide-1 (GLP-1) compared to casein [94]. Interestingly, this effect may be driven by the inhibitory role that whey protein has on dipeptidyl peptidase IV, the enzyme responsible for the degradation of active GLP-1 [95]. By preventing the degradation of systemic GLP-1, more GLP-1 remains active in the circulation and has a more protracted effect on satiety. Hall et al. [96] also demonstrated greater plasma elevations of cholecystokinin (CCK), another satiety-inducing hormone secreted by the gut, after whey protein consumption. An elevated circulating CCK with consumption of whey protein [96] may be due to the presence of glycomacropeptide (GMP) promoting the secretion of CCK [97]. In support of the unique ability of GMP to stimulate systemic CCK release, Veldhorst and colleagues [98] demonstrated an increased energy intake at lunch following the consumption of a meal containing GMP-depleted whey protein compared to a ‘GMP-replete’ whey protein.

When measurement periods are prolonged and ad libitum food intake is assessed after several hours, casein appears to be mildly better than whey protein for appetite suppression [99]. Indeed, casein supplementation was shown in one study to reduce energy intake relative to whey supplementation over a seven day long supplementation trial [100]. Importantly, the effects of individual protein sources on satiety may be equivalent if protein intakes are high enough. Veldhorst et al. [94] found that a whey protein-containing breakfast delivering 10% protein, 55% carbohydrate, and 30% fat more effectively suppressed energy intake at lunch when compared to a casein protein-containing breakfast with the same macronutrient profile. When the percentage of meal-derived protein was increased to 25% (with a corresponding reduction in fat), there were no observable differences in lunch meal energy intake between casein and whey protein groups [94]. Given that athletes and recreationally active adults require higher amounts of protein to maintain LBM during energy restriction, the differences observed between whey and casein at smaller relative doses will likely be diluted when protein intake is increased. Therefore, akin to athletes in energy balance, athletes subjected to energy-restricted diets should focus primarily on fulfilling daily protein requirements (1.6–2.4 g/kg/day) and only then focusing on other relevant variables of protein supplementation (i.e., protein source and timing).

## 5. Practical Recommendations

We provide here some translatable messages that summarize some of the more salient points we have made in this review.

### 5.1. Individuals in Energy Balance

1. Consume ~0.4 g/kg body mass (i.e., 0.24 plus 0.06 with protein added to account for the influence of other macronutrients in meals and protein quality), to maximally stimulate MPS following a period of rest or exhaustive resistance exercise.
2. Spacing protein-containing meals ~3–5 h throughout the day maximizes MPS rates over the course of a 12 h (i.e., waking) period.
3. Practice pre-sleep protein ingestion (1–3 h prior to sleep) to offset declines in MPS that would occur during an overnight fasting period.
4. To maximize muscle protein accretion with resistance exercise, daily protein intakes should be ~1.6 g/kg/day and up to 2.2 g/kg/day. This intake can be achieved by ingesting 3 meals, each containing ~0.53 g/kg protein, or 4 meals containing ~0.4g/kg protein.

### 5.2. Individuals in Energy Restriction

1. Daily protein requirements are greater than they are during period of energy balance to promote the maintenance or increase in lean body mass.
2. Resistance exercise should be performed during energy restriction to promote the retention of lean body mass if desired.
3. For athletes ‘cutting’ weight over an extended period, high quality protein sources such as whey and casein, or a blend of each, should be chosen to optimize appetite control and ensure dietary compliance.
4. To promote lean body mass retention during weight loss, protein intakes of ~2.3–3.1 g/kg/day have been advocated. Exercise-naive adults who have a greater body fat percentage should aim to achieve the lower end of this range, whereas leaner individuals with resistance-training experience who are more vulnerable to losing lean body mass during energy restriction might aim for the higher end of this range.

## 6. Summary and Conclusions

The human body is capable of digesting large quantities of dietary protein. However, not all the constituent amino acids are utilized by the translational machinery to synthesize new proteins. With consumption of an isolated protein source, beyond a protein intake of ~0.3 g/kg body mass (i.e., 0.24 plus the upper-end of the 95% CI), MPS is saturated and the rate of amino acid catabolism through oxidation and urea production increases and so less amino acids are available for protein synthesis. Individuals performing whole-body resistance exercise may require larger protein doses to maximize the anabolic effects of protein, yet these effects are only marginally greater than what is observed at 20 g protein. Given that the muscle becomes refractory to the presence of amino acids, such that MPS returns to basal levels after ~3 h despite sustained hyperaminoacidemia, protein meals should be separated by ~3–5 h to maximize MPS over the waking period. While these strategies have proven to be most effective in acute settings (i.e., over a 12 h capture period), the most salient variable determining the effectiveness of protein supplementation on gains in muscle size during resistance training is still total daily protein intake. In a large meta-analysis, protein intake was shown to promote additional gains in lean body mass beyond those observed with resistance exercise alone; however, beyond a daily intake of 1.6 g/kg body mass per day (up to as high as 2.2 g/kg/day), the additional effects of protein are greatly diminished. Rather than further stimulating MPS, large intakes of protein beyond what we are recommending may modulate anabolism by suppressing proteolysis; however, we lack experimental evidence for this in muscle. We caution against strategies that focus on suppressing MPB as we contend that efficient removal of damaged proteins would require a robust and fully functional proteolytic response. We are unaware of any potential improvements with respect to skeletal muscle hypertrophy by strategies that suppress MPB. Thus, athletes in energy-balance seeking to optimize the adaptive potential of their resistance-training programs are advised to first ensure that they are consuming ~1.6 g/kg body mass per day of protein, and tailor their dosing strategies to meet this overarching goal.

Periods of energy restriction result in significant reductions of lean body mass. Lean individuals, and those with more training experience, appear to be more susceptible to reductions in skeletal muscle size relative to heavier, training-naïve individuals. Reductions in LBM are primarily driven by reductions in postabsorptive rates of MPS and a reduced sensitivity to the presence of a protein bolus. To effectively prevent these declines in MPS during both postabsorptive and postprandial periods, daily protein intake have been recommended to be increased to ~2.3–3.1 g/kg/day, and leaner athletes may wish to aim for intakes at the higher end of this range. Participation in resistance exercise is sufficient to counteract the decrements observed in MPS following an acute period of energy restriction and, when paired with sufficient protein intake, can result in gains in LBM. Thus, this combination of sufficient protein intake and performing regular resistance exercise should form the cornerstone of any weight-loss diet. Athletes who are attempting to cut body mass for a competition several weeks in advance can enhance the satiating effects of each meal, and thus dietary compliance, by ensuring that high quality protein is ingested. During acute periods, whey protein has greater appetite suppressive effects than casein protein, however the situation is reversed when measurements are obtained several hours after food intake. Thus, an athlete may wish to consume a blend of proteins in addition to other whole-food sources to achieve better satiety. However, such a strategy may be unnecessary if protein intakes approach those required to maintain skeletal muscle mass during periods of energy restriction.
